# ﻿A new tree species of *Compsoneura* (Myristicaceae) from the Andean forests on the Eastern Cordillera Range, Colombia

**DOI:** 10.3897/phytokeys.251.136715

**Published:** 2025-01-27

**Authors:** Boris Villanueva-Tamayo, Carlos Paz-López, William Ariza-Cortés

**Affiliations:** 1 Jardín Botánico de Bogotá “José Celestino Mutis”, Av. José Celestino Mutis #68-95, Bogotá, Colombia; 2 Herbario Forestal (UDBC), Universidad Distrital Francisco José de Caldas, Carrera 5 Este No. 15-82, Bogotá, Colombia; 3 Grupo de Investigación Uso y Conservación de la Diversidad Forestal, Universidad Distrital Francisco José de Caldas, Carrera 5 Este No. 15-82, Bogotá, Colombia

**Keywords:** Dendritic trichomes, endemism, *Eucompsoneura* section, Sub-Andean forests, Bosques subandinos, Endemismo, Sección *Eucompsoneura*, Tricomas dendríticos

## Abstract

*Compsoneuracrassitepala*, a new species of Myristicaceae is described, illustrated and its morphological relationships with related species are discussed. This new species is found in Andean forests between 1400 and 1900 m a.s.l., located in the mountainous area of the Magdalena River Basin, Department of Boyacá, Colombia. *Compsoneuracrassitepala* closely resembles *Compsoneuralapidiflora* in having a thick perianth in pistillate flowers. However, it differs by its leaves, which exhibit weak and partially brochidodromous venation with dendritic trichomes featuring a single axis on the underside. Additionally, it has floral characteristics such as a zig-zag pattern in the rachis direction of inflorescence and unusually thick, fleshy tepals in staminate flowers. Moreover, *C.crassitepala* is a remarkable species recorded at higher altitudes in Colombia, in an otherwise predominantly lowland forests genus (below 1550 m). A taxonomic key for the identification of the species of the genus is provided.

## Abstract (es)

Se describe e ilustra *Compsoneuracrassitepala*, una especie nueva de Myristicaceae, y se discuten sus relaciones morfológicas con especies afines. Esta nueva especie se encuentra en bosques andinos entre 1400–1900 m s.n.m., localizados en la zona montañosa de la cuenca del río Magdalena, departamento de Boyacá, Colombia. *Compsoneuracrassitepala* se asemeja a *Compsoneuralapidiflora* por tener un perianto grueso en las flores pistiladas. Sin embargo, se diferencia por sus hojas, que exhiben una venación débil y parcialmente brochidódroma con tricomas dendríticos que presentan un solo eje en el envés. Además, presenta características florales como un patrón en zig-zag en la dirección del raquis de la inflorescencia y tépalos inusualmente gruesos y carnosos en las flores estaminadas. Además, *C.crassitepala* es una especie particularmente registrada a altitudes más altas en Colombia, en un género predominantemente de bosques de tierras bajas. (por debajo de 1550 m). Se proporciona una clave taxonómica para la identificación de las especies del género.

## ﻿Introduction

Myristicaceae R. Brown consists of trees, shrubs and less frequent lianas (i.e. *Pycnanthus* Warb.) and comprises 21 genera and approximately 510 species with a pantropical distribution (Kühn and Kubitzki 1993; Sauquet 2004). In America are found ca. 110 species (Jaramillo-Vivanco and Balslev 2020) in six genera: *Bicuiba* W. J. de Wilde, *Compsoneura* Warb., *Iryanthera* (A. DC.) Warb., *Osteophloeum* Warb., *Otoba* (A. DC.) H. Karst and *Virola* Aubl., which are mainly distributed in wet forests from southern Mexico to Bolivia (in the Amazon Basin, Chocó and Central America), the Lesser Antilles and Matto Grosso and Santa Catharina States in southern Brazil (Jaramillo-Vivanco and Balslev 2020).

Myristicaceae can be easily identified by their vegetative characteristics, which include reddish, yellowish, orange, amber (in Osteophloeum), translucent, astringentand sometimes aromatic exudates that emerge when the bark is cut or the branches are broken (Wilson 2004; Sasaki 2009; Aymard et al. 2020). The leaves are simple, alternate, distichous, entire and without stipules; the pubescence of petioles, branches, twigs and leaf lamina, is composed of stellate, sessile, substipitate or stipitate, dendritic or 2-branched (malpighiaceous), persistent or evanescent trichomes (Santamaría-Aguilar et al. 2019; Vásquez-Martínez and Soto-Shareva 2019). Additionally, one notable characteristic all family members exhibit is a unique growth pattern, represented by the architectural model known as “Massart” (Hallé et al. 1978). This pattern of tree architecture is characterised by a monopodial stem and a rhythmic growth of the lateral branches, which extend (almost or completely) horizontally (plagiotropy) (Hallé et al. 1978).

In the last two decades, significant progress has been made in the discovery of new species and in enhancing our understanding of the distributions within the genus Compsoneura (A. DC.) Warb., *Otoba* (A. DC.) H. Karst. and *Virola* Aubl (Santamaría-Aguilar et al. 2019; Santamaría-Aguilar and Lagomarsino 2021). Amongst the most remarkable contributions is the systematic study of *Compsoneura* by Janovec (2000), his monograph is an important work for studies in this genus. Other contributions are: Jaramillo-Vivanco and Balslev (2001), Janovec and Neill (2003), Vásquez-Martínez and Soto-Shareva (2019), Santamaría-Aguilar et al. (2019), Jaramillo-Vivanco and Balslev (2020), Santamaría-Aguilar and Lagomarsino (2021), Frost et al. (2022), Ríos Paredes et al. (2023) and a new species of *Compsoneura* from interandean valleys of middle Magdalena and lower Cauca Rivers (Villanueva-Tamayo and Cogollo Pacheco 2024).

The genus *Compsoneura* is distinguished from the other genera of the family by its leaves with conspicuous and prominent tertiary veins, subparallel to nearly perpendicular to perpendicular in the primary and secondary veins (Gentry 1993; Janovec 2000). Fruits with two longitudinally opening valves, pericarp thin or thick to woody, with carinate or smooth surface, the aril rudimentary, short or deeply laciniate or entire (Rodrigues et al. 2001; Aymard et al. 2020). *Compsoneura* is distributed in the Neotropics, from southern Mexico to the Amazon Basin (Jaramillo-Vivanco and Balslev 2001). Currently, 18 species are recognised, of which 12 are present in Colombia (Villanueva-Tamayo and Cogollo Pacheco 2024). The Colombian species are distinctly localised: *Compsoneuraatopa*, *C.cuatrecasasii*, *C.rigidifolia* and *C.trianae* inhabit the western Colombian Pacific Forests. Meanwhile, *Compsoneuramutisii*, *C.choibo*, *C.anoriensis*, *C.claroensis* and the newly-described species *C.crassitepala* are found in the northern Andean regions of Colombia. *Compsoneuracapitellata* and C. *sprucei* are widely distributed. While *C.debilis* is restricted to white-sand forests in the Amazon Basin and *C.schultesiana* has been found exclusively in the Colombian Amazon (Janovec 2000; Villanueva-Tamayo and Cogollo Pacheco 2024).

Here, a new species of Compsoneura is described and illustrated. This fascinating discovery was made during botanical fieldwork in the montane forests of the middle basin of the Magdalena River, in the Boyacá Department of Colombia

## ﻿Materials and methods

The new species was first collected during the floristic characterization conducted as part of the forest management plans in the western region of the Boyacá Department. The specimens were recollected with fruits and staminate flowers in June 2022. Additional material with pistillate flowers, staminate flowers and fruits were recollected in July 2023.

Based on the review for the publication of *Compsoneurachoibo* Villanueva & Cogollo, specimens were examined in the herbaria best represented in *Compsoneura* collections in Colombia (COL, HUA, JAUM, UDBC), locating some type specimens that had not been separated until now. A database was consolidated that allowed the identification of important morphological characters (veins, fruit testa) and the development of a taxonomic key published in Villanueva-Tamayo and Cogollo Pacheco (2024), where, for the first time, a key based on morphological characters is proposed, supported by reproductive elements and with relevant information on their distribution and ecology. We propose to enhance this key by incorporating this new species.

The botanical specimens of this new species of *Compsoneura* were compared with collections from some of the principal herbaria in Colombia: BOG, COAH, COL, FMB, HUA, JBB, MEDEL, TOLI and UDBC (acronyms follow Thiers (2024), continuously updated) and analysed with species key published by Villanueva-Tamayo and Cogollo Pacheco (2024). For the selection of the species to be compared, characteristics such as leaf venation, indumentum, inflorescence type, perianth characteristics, fruit (surface area) and geographic location were taken into account and compared with nomenclatural types available on COL Herbarium, Global Plants on JSTOR (https://plants.jstor.org/) and Tropicos (https://tropicos.org/home). Only the recently published *Compsoneuranallarettiana* lacks a registered image of its type; however, it is accompanied by a detailed description.

The specialized literature on *Compsoneura* and related genera was reviewed, mainly Warburg (1897; 1905), Smith and Wodehouse (1937), Smith (1938, 1950, 1956), Janovec (2000), Jaramillo-Vivanco and Balslev (2001), Janovec and Harrison (2002), Janovec and Neill (2003), Jaramillo-Vivanco and Balslev (2020)Santamaría-Aguilar and Lagomarsino (2021); Ríos Paredes et al. (2023) and Villanueva-Tamayo and Cogollo Pacheco (2024).

The morphological measurements were taken using an Ubermann digital caliper. Images of various plant parts, including branches, leaves and inflorescences, were captured using a SONY Alpha 7 II camera with a 50 mm lens and Raynox super macro DCR–250. The illustration was freehand-drawn on an iPad Pro and Lankester plate was prepared, based on images taken in the field. The preliminary conservation status was assessed according to IUCN categories and criteria (IUCN 2024). The extent of occurrence (EOO) and area of occupancy (AOO) were calculated using the Geospatial Conservation Assessment Tool (GeoCat) (Bachman et al. 2011), which is continuously updated through https://geocat.kew.org/.

## ﻿Taxonomic treatment

### ﻿ Compsoneura crassitepala

Taxon classificationPlantaeMagnolialesMyristicaceae

Villanueva, Paz-López & W.Ariza sp. nov.

7C7C234D-44D7-5FFC-8D69-DB94BDCDD3DF

urn:lsid:ipni.org:names:77355777-1

Figs 1 , 2

#### Type.

Colombia • Boyacá: Municipio de Coper, Vereda Cucunubá, finca El Brasil, 5°24'47.0"N, 74°02'39.00 W, 1720 m a.s.l., 29 July 2023 (fl ♂), *Carlos A. Paz et al.* 2158 (holotype: UDBC!, barcode no. 49999; isotypes: COL!, FMB!, JAUM!, JBB!).

#### Diagnosis.

This new species can be distinguished from other species by its staminate and pistillate flowers, which have a spherical and fleshy perianth. It is similar to *C.nallarettiana* in its weakly brochidodromous secondary venation (only the first two veins are non-anastomosed) and differs in pedunculate fruits (vs. sessile fruits) and leaf blades oblong or elliptic-oblong (vs. leaf blades elliptic) and is similar to *C.cuatrecasasii* in slightly brochidodromous secondary venation and trichomes minute 2-branched. However, it differs in leaf oblong or elliptic-oblong (vs. leaf blades obovate to orbiculate), abaxial lamina densely ferruginous tomentose (vs. abaxial lamina glabrescent to glabrous when mature) leaf blade coppery when dry (vs. leaf blades that dry red-brown).

**Figure 1.**
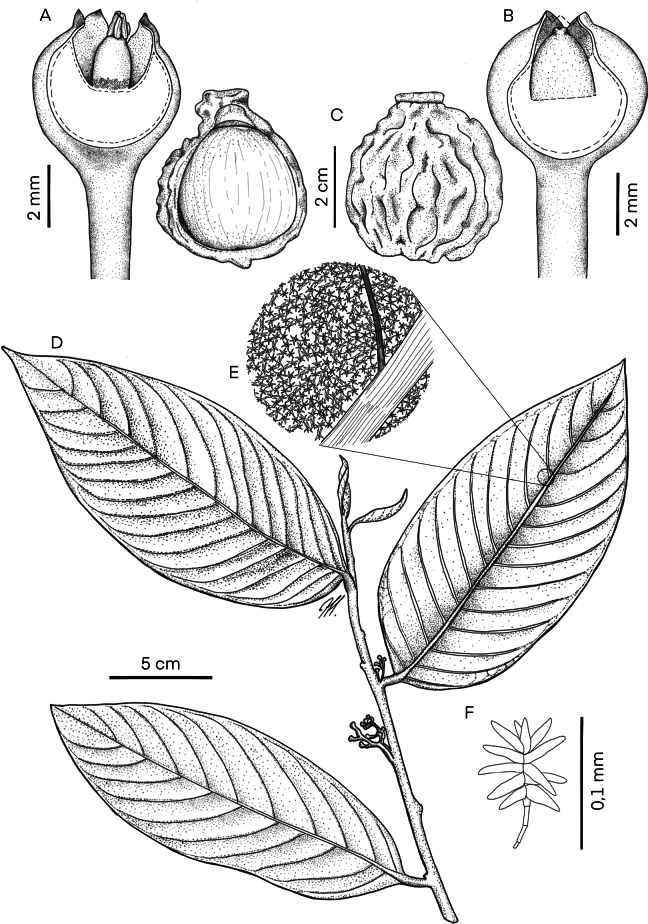
*Compsoneuracrassitepala***A** staminate flower **B** pistillate flower **C** fruit without pericarp showing the seed with one valve removed (left), fruit entire (right) **D** terminal branch with a pistillate inflorescence showing two leaves on the upper surface and one leaf on the lower surface (*B. Villanueva-T et al. 6734*) **E** trichomes on abaxial leaf blade **F** trichome (*B. Villanueva-T et al. 6734*). Illustration by Manuela Sánchez (JBB).

#### Description.

Dioecious tree, 18 m tall, pyramidal crown, rhytidome slightly fissured and detachable in plates, the inner bark exuding sap red-hyaline; wood red-cream colour; branchlets ferruginous when dry, 5.3–11.3 mm wide, tomentose with dendritic trichomes, when young densely ferruginous tomentellous, hairs dendritic trichomes with only one axis, densely brown ferruginous when old, densely covered with lenticels, 0.7–1.6 × 0.5–0.7 mm. ***Leaves*** simple, alternate; ***petioles*** cylindrical stout, light ferruginous when fresh or coppery when dry, 12.0–13.4 × 4.1–4.8 mm; ***leaf blades*** coriaceous or strongly coriaceous when dried, oblong or elliptic-oblong, 21.0–25.5 × 6.5–8.2 cm, width at ¼ length 5.5–7.0 cm, width at ½ length 6.5–8.1 cm, width at ¾ length 5–6.2 cm; base rounded or obtuse or slightly attenuate, not revolute; apex acute, acuminate or apiculate, acumen 4.5–8.1 mm; margin entire; ***adaxial*** lamina surface shiny, light green or yellow-green when fresh, coppery when dry, with dispersed dendritic trichomes in young leaves; ***abaxial*** lamina surface dull, densely ferruginous tomentose, dendritic trichomes, light orange when fresh, yellowish-brown when dry; costa raised above near the base, scattered dendritic trichomes on young leaves and at the base of older leaves, raised; secondary veins 11–16 per side, spaced 6–16 mm, slight arcuate ascending, the first two veins not anastomosing completely or only between, the next veins strongly brochidodromus. ***Staminate inflorescences***: 3.1–6.1 × 1.31–2.6 cm long, rachis direction in a zig-zag pattern, axillary, 1–2-paniculate, densely ferruginous tomentellous with dendritic trichomes, peduncle 5.2–13.3 × 1.7–2.7 mm, main axes with 3–8 ramifications, alternate. ***Staminate flower buds***: subglobose densely ferruginous tomentose. ***Staminate flowers***: subglobose or obovoid-globose, in fascicles of 4–8 flowers, fleshy when fresh and dry, densely ferruginous tomentellous, with dendritic trichomes on the outer side, thinly ferruginous, covered by dendritic trichomes inside, borne on a receptacle 1.5–3.4 mm wide; pedicels 0.5–0.6 × 0.1–0.12 cm; perianth 3.9–4.6 × 3.6–4.5 mm, globose; lobes 3–4, fleshy, erect, triangular or broadly triangular, apex reflexed when mature, 1.5–1.8 × 1.9–2.4 mm, connate by ⅔; ***androecium***: 1.8–2.3 mm long, filament column 0.9–2.1 × 0.6–1.8 mm, with a ferruginous, tomentose ring of dendritic trichomes at the base, anthers 6, dorsally adnate by ⅓, strongly erect ascending from base to apex 0.7–1.1 × 0.2–0.3 mm obtuse to apex. ***Pistillate inflorescences***: 1.9–2.1 × 1.5–1.6 cm, rachis direction in a zig-zag pattern, axillary, 1–2 paniculate, densely ferruginous tomentellous with dendritic trichomes, peduncle 5.2–7.4 × 2.3–3.2 mm, main axes with 1–2 ramifications, alternate. ***Pistillate flower buds***: subglobose densely ferruginous tomentose. ***Pistillate flowers***: subglobose or obovoid-globose, in fascicles of 4–5 flowers, fleshy when fresh and dry, densely ferruginous tomentellous, with dendritic trichomes on the outer side, thinly ferruginous, covered by dendritic trichomes inside; borne on a receptacle 2.5–3.1 mm wide, pedicels 0.1–0.5 × 0.07–0.2 cm; perianth fleshy when fresh and dry, 5.3–5.7 × 5.5–5.9 mm, lobes 3–4, fleshy, erect, triangular or broadly triangular, with lobes slightly curved at apex at maturity, 0.8–1.3 × 0.6–1.3 mm, connate by ⅓, densely ferruginous tomentellous with dendritic trichomes in both sides; ovary, ovate, tannish-brown, cover by densely ferruginous tomentellous, fresh or dry, 2.8–3.7 × 2.2–2.8 mm; stigma bilobed, dark brown, 0.4–0.9 × 0.3–0.8 mm. Infructescences axillary, solitary or paired, 5–8.5 cm long; peduncle 11.2–11.9 × 3.3–5.3 mm, terete or subterete, with short tomentellous dendritic trichomes; fruits 1–2 per infructescence. ***Fruits*** rounded, brown when fresh, with the surface strongly and irregularly sulcate, 4.3–6.2 × 4.2–6.2 cm, grooves 1.0–5.0 mm deep, 0.2–0.5 mm wide, thinly ferruginous, with dendritic trichomes outside, dehiscent; seed (1), 3.3–4.5 × 3.3–4.1 cm, testa minutely reticulate, shiny, brown, aril thin and lignified when mature, pale brown fresh or dry, lacinate, reduced, 0.3–0.5 cm long.

**Figure 2.**
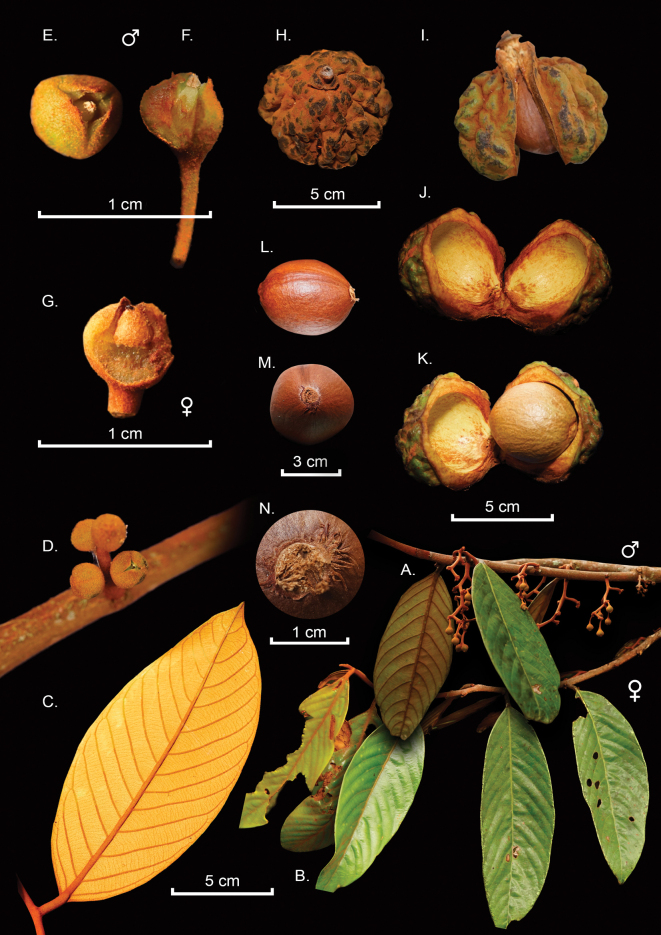
*Compsoneuracrassitepala***A** branches with staminate inflorescences and leaves showing both faces *(C. Paz 2158* UDBC) **B** terminal branch bearing pistillate inflorescence (*B Villanueva-T. et al. 6734*) **C** underside of ferruginous leaf (*B Villanueva-T. et al. 6734*) **D** pistillate inflorescence (*B Villanueva-T. et al. 6734*) **E** staminate flower *(C. Paz 2158* UDBC) **F** androecium visible by side cut *(C. Paz 2158* UDBC) **G** pistil, showing the thick perianth (*B Villanueva-T. et al. 6734*) **H** fruit sulcate, densely ferruginous pubescent with stellate trichomes and vestigial tepals, showing the sulcate surface (*B Villanueva-T et al. 6734*) **I** external view of open pericarp, showing the sulcate surface (*B Villanueva-T et al. 6734*) **J** internal view of open pericarp (*B Villanueva-T et al. 6734*) **K** open fruit showing seed and pericarp thickness (*B Villanueva-T et al. 6734*) **L** seed side view without aril (*B Villanueva-T et al. 6734*) **M** seed without aril from above (*B Villanueva-T et al. 6734*) **N** detail of the thin and lignified aril (*B Villanueva-T et al. 6734*). Photos by Boris Villanueva-T., Lankester plate by Daniel Amaya-Jiménez.

#### Distribution and habitat.

**Figure 3.**
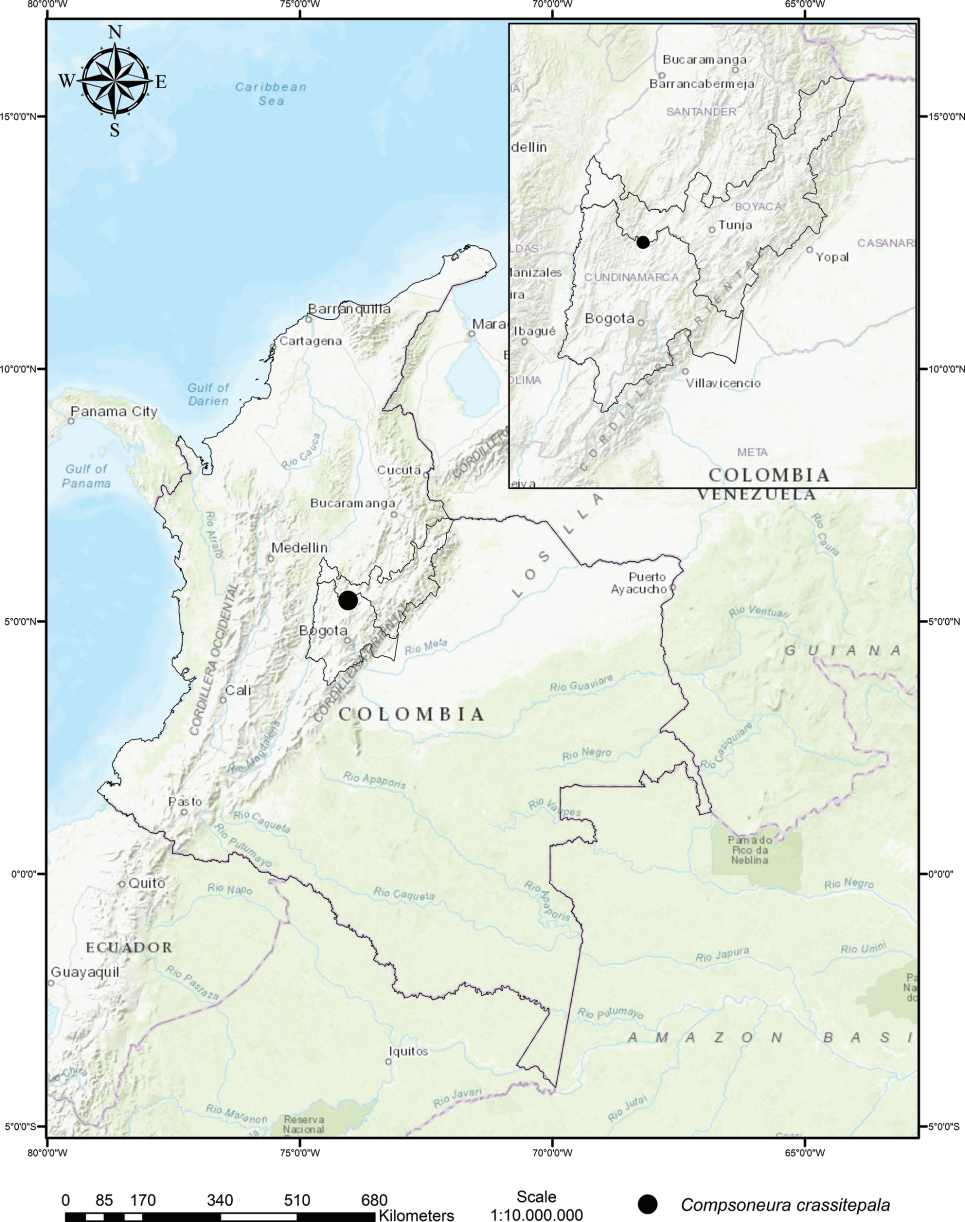
Distribution map of *Compsoneuracrassitepala*.

According to Cuatrecasas (1958), this species inhabits the sub-Andean forests of the Eastern Cordillera in the Boyacá Department, at elevations between 1500 and 1900 m. It is endemic to these forests, where it is associated with *Dictyocaryumlamarckianum* (Mart.) H. Wendl. (Arecaceae), *Conceveibapleiostemona* Donn. Sm. (Euphorbiaceae) and *Andesanthuslepidotus* (Bonpl.) P.J.F. Guim. & Michelang. (Melastomataceae) (Fig. 3).

*Compsoneuracrassitepala* has been recorded in sub-Andean forest fragments within pasturelands, at the boundary of the Boyacá and Cundinamarca Departments, on the western slopes of the Eastern Cordillera in Colombia. This new species is sympatric with *C.rigidifolia*, the species recorded at the highest altitude in Colombia.

#### Additional specimens examined.

Colombia • Boyacá: Coper, Vereda Cucunubá Finca El Brasil, 1700 m a.s.l., 5°24'24.65"N, 74°02'53.58"W, 21 Jun 2022 (♀ fl, fr), *M. Betancour et al. 51* (UDBC!); • Ibid, 5°24'24.27"N, 74°02'53.63"W, 1700 m a.s.l., 22 Jun 2022 (♂ fl), *M. Betancour et al. 58* (JBB!, UDBC!); • Ibid, 5°24'41.5"N, 74°02'13"W, 1582 m a.s.l., 29 Jul 2023 (♀ fl), *B. Villanueva-T. et al. 6734* (JBB!, barcode no. 40030); • Ibid, 5°24'42"N, 74°02'12"W, 1575 m a.s.l., 29 Jul 2023 (♂ fl), *B. Villanueva-T. et al. 6735* (JBB!, barcode nos. 40031 & 40032); • Ibid, 5°24'47.0"N, 74°02'39.00 W, 1769 m a.s.l., 29 Jul 2023 (♀ fl, fr), *W. Ariza et al. 10016* (COL!, FMB!, JAUM!, JBB!, UDBC!); • Ibid, 5°24'48.80"N, 74°02'35.70"W, 1729 m a.s.l., 29 Jul 2023 (♂ fl) *A. Torrejano-M et al. 1356* (JBB!, UDBC!); • Ibid, 5°24'46.96"N, 74°02'35.70"W, 1735 m a.s.l., 16 Mar 2024 (fr) *B. Villanueva-T. et al. 6840* (JBB!, UDBC!).

#### Phenology.

Collected with staminate and pistillate flowers, and fruit during June, July and fruit in March.

#### Conservation status.

*Compsoneuracrassitepala* has an area of occupancy (AOO) of 1 km^2^ and an extent of occurrence (EOO) of approximately 4,200 km^2^ and, according to the IUCN criteria (IUCN 2024), it should be considered “Endangered” (EN) (B1ab). This area encompasses a mosaic of pastures with exploited forests and limited cultivation. Currently, there are no protected areas or policies that support the conservation of the species’ habitat.

#### Etymology.

The specific epithet of this species refers to its unusually thick and fleshy tepals.

#### Common names.

Sangrino (Colombia, Boyacá; *C. Paz et al. 2158*).

#### Discussion.

Based on different diagnostic characteristics such as secondary vein, drying colour of leaf blades and pericarps, Janovec (2000) proposed to divide the genus *Compsoneura* into two sections. He redefined the classic study of Warburg in 1897, but these sections are not formally published. Other species published after 2000 (i.e. *C.choibo* and *C.lapidiflora*) do not fit within the two informally proposed sections and, for the last species, its fruit is unknown. Therefore, we prefer to classify the new species within Warburg’s classic concept of the *Eucompsoneura* section, due to its partially fixed anthers to the filament (Warburg 1897, 1905).

*Compsoneuracrassitepala* shows a combination of characters that differentiate it from the other species in the genus. The thin and lignified aril not previously recorded in *Compsoneura* (Fig. 2N), the rachis in staminate inflorescence in a zig-zag pattern (Fig. 2A) and the subglobose or obovoid-globose and fleshy perianth in staminate and pistillate flowers (Fig. 2F, G).

This species shares characteristics with *Compsoneuranallarettiana*, such as the slightly brochidodromous secondary venation and the anastomosis from the second vein. However, *C.crassitepala* can be distinguished by its pedunculate fruits (vs. sessile fruits) and elliptic-oblong leaf blades (vs. elliptic or elliptic-ovate leaves). The new species shares the following characters with *C.cuatrecasasii*: oblong or elliptic-oblong leaves and slightly brochidodromous secondary venation. *C.crassitepala* differs from *C.cuatrecasasii* by the leaf blades that dry yellowish-brown and have an abaxial lamina densely ferruginous tomentose with dendritic trichomes (vs. leaf blades that dry red-brown and are glabrescent to glabrous when mature on the abaxial lamina with minute 2-branched trichomes). Additionally, after the revision of herbarium specimens, based on morphological traits such as leaf blade veins and size, perianth lobes and pericarp texture and following the process of Villanueva-Tamayo and Cogollo-Pacheco (2024), an identification key for the species of the genus is provided.

### ﻿Key to the species of *Compsoneura*

Based in Villanueva-Tamayo and Cogollo-Pacheco (2024).

Expanded Key from the review by Villanueva-Tamayo and Cogollo-Pacheco (2024).

**Table:** 

1	Leaf blades secondary venation brochidodromous	**2**
–	Leaf blades secondary venation eucamptodromous	**10**
2	Leaf blades elliptic, elliptic-ovate or obovate-elliptic, 15–35 cm long	**3**
–	Leaf blades oblong, elliptic-oblong or obovate, if elliptic, longer than 40 cm long	**6**
3	Fruits with a thin pericarp, less than 1.5 mm thick, slightly carinate on one side, with 9–10 subsidiary longitudinal costae (irregularly ridged); Pacific Forests of Panama, Colombia and Ecuador	***C.rigidifolia***
–	Fruits with a pericarp thickness, equal to or greater than 2 mm, strongly carinate, with fewer than 9 irregular and thick costae; Andean Region of Colombia, Amazonian Ecuador and Peru	**4**
4	Secondary venation slightly brochidodromous, anastomosed from the second vein; fruits sessile	***C.nallarettiana***
–	Secondary venation strongly brochidodromous; fruits pedicellate	**5**
5	Leaf blades and perianth covered by dendritic trichomes, sepals thick, extremely rigid, perianth lobes cucullate. Amazonian Ecuador (250–400 m a.s.l.)	***C.lapidiflora***
–	Leaf blades and perianth glabrous or glabrescent throughout, the trichomes minute, sessile or short-stalked, 2-branched, not dendritic; sepals thin, not extremely rigid, perianth lobes erect and elongated; foothills and Andean Ecuador and Peru (600–2160 m a.s.l.)	***C.diazii***
6	Leaf blades 40–50 cm long, oblong to oblong-elliptic; mid-rib and secondary veins covered by ferruginous stellate trichomes on the lower surface; Colombia Pacific coast	***C.atopa***
–	Leaf blades less than 35 cm long, oblong to oblong-elliptic or obovate; mid-rib and secondary veins glabrescent to sparsely tomentellous on the abaxial lamina; northern Colombian interandean valleys and Colombia Pacific coast	**7**
7	Leaf blades obovate to orbiculate, glabrescent to ferruginous-tomentellous when young, glabrescent to glabrous when mature on the abaxial lamina; trichomes minute 2-branched, drying red brown; Colombian Pacific coast	***C.cuatrecasasii***
–	Leaf blades oblong to oblong-elliptic, densely ferruginous tomentose, dendritic trichomes, glabrescent to sparsely tomentellous on the lower surface, northern Andean Colombian	**8**
8	The first two veins not anastomosing completely, leaf blades drying coppery, abaxial lamina densely ferruginous tomentose with dendritic trichomes, northern Andean Colombian to 1500 m a.s.l.	***C.crassitepala***
–	Strongly brochidodromous secondary venation, leaf drying greyish-brown or brown to dark brown, abaxial lamina glabrescent to sparsely tomentellous with trichomes minute stellate 3–4 branched, northern Colombian interandean valleys below 1000 m a.s.l.	**9**
9	Leaf lamina drying greyishbrown, fruit with longitudinal ridges giving rise to horn-like projections; Anori River Valley (northern Colombian Andes)	***C.anoriensis***
–	Leaf lamina drying brown to dark brown; fruit with longitudinal ridges, lacking horn-like projections; middle Magdalena River Valley	***C.claroensis***
10	Fruits carinate, leaves sparsely tomentellous on the abaxial surface at the base (Panama, Colombia, Brazil, Ecuador and Peru)	***C.capitellata***
–	Fruits non-carinate, leaves glabrous at base (Bolivia, Brazil, Ecuador, Peru, Colombia and Venezuela)	**11**
11	Leaves coriaceous to thick coriaceous, staminate inflorescence reduced to a tightly clustered axillary panicle, ≤ 1.3 cm long; restricted to white sand ecosystems of the Rio Negro Basin (NW Brazil, SE Colombia)	***C.debilis***
–	Leaves membranaceous to thin coriaceous, staminate inflorescence well-developed, not reduced, exceeding 1.3 cm in length; Found in lowland forests of Central and South America, not restricted to white sand ecosystems of the Rio Negro Basin (NW Brazil, SE Colombia)	**12**
12	Staminate inflorescence a single raceme	**13**
–	Staminate inflorescence paniculate	**14**
13	Leaves thin, less than 6 secondary veins when mature; (Brazil)	***C.racemosa***
–	Leaves chartaceous, with more than 6 secondary veins when mature; Amazon Basin of Bolivia, Brazil and Ecuador	***C.ulei***
14	Staminate flower buds obovate	**15**
–	Staminate flower buds ovate	**17**
15	Staminate inflorescences 4–9 cm long; perianth often 4-lobed; flowers 2 or 3 per fascicle, those towards apex of inflorescence solitary; filament column carnose or spongiose, swollen distally and leading into a carnose obconical connective; Costa Rica, Honduras and Panama	***C.excelsa***
–	Staminate inflorescences 2–4 cm long; perianth 3-lobed; flowers 3–7 per fascicle; filament column not carnose or spongiose, not swollen distally; Colombia	**16**
16	Leaf blades papyraceous, usually translucent, slightly obovate, the apex abruptly acuminate; Colombian interandean valleys	***C.mutisii***
–	Leaf blades thick coriaceous, opaque, ovate, ovate-elliptic or elliptic, the apex cuspidate; Colombian Pacific coast	***C.trianae***
17	Fruit densely pubescent ferruginous or ferruginous tomentulose, the pericarp strong and ligneous; of Colombian interandean valleys	***C.choibo***
–	Fruit glabrous, the pericarp thin, non-ligneous, smooth	**18**
18	Shrubs, treelets or small trees; leaf blades thick coriaceous, strongly translucent; Colombian Amazon Basin	***C.schultesiana***
–	Trees; leaf blades chartaceous or papyraceous, slightly translucent	**19**
19	Perianth vasiform, lobes erect; leaf blades elliptic, elliptic-obovate; stem tan, striate not exfoliating; Central America	***C.mexicana***
–	Perianth cupuliform, urceolate, lobes recurved at apex; leaf blades elliptic to elliptic-ovate; stem brown or darker black, bark exfoliating; Amazon Basin of Brazil, Colombia, Ecuador, Peru and Venezuela	***C.sprucei***

## Supplementary Material

XML Treatment for Compsoneura crassitepala

## ﻿Additional information

### Author ORCIDs

Boris Villanueva-Tamayo https://orcid.org/0000-0002-6929-3572

Carlos Paz-López https://orcid.org/0009-0000-0071-2380

William Ariza-Cortés https://orcid.org/0000-0002-8423-8256

### Data availability

All of the data that support the findings of this study are available in the main text.

## References

[B1] Aymard GA Castro-Lima F Arellano PH (2020) Identificación de Myristicaceae de la cuenca del Río Negro en ausencia de flores y frutos. Pittieria 44: 28–55.

[B2] Bachman S Moat J Hill AW de la Torre J Scott B (2011) Supporting Red List threat assessments with GeoCAT: Geospatial conservation assessment tool. ZooKeys 150: 117–126. 10.3897/zookeys.150.2109 PMC3234434 22207809

[B3] Cuatrecasas J (1958) Aspectos de la vegetación natural de Colombia. Revista de la Academia Colombiana de Ciencias Exactas, Físicas y Naturales 10(1): 221–268. 10.18257/raccefyn.570

[B4] Frost L Santamaría‐Aguilar D Singletary D Lagomarsino LP (2022) Neotropical niche evolution of Otoba trees in the context of global biogeography of the nutmeg family. Journal of Biogeography 49(1): 156–170. 10.1111/jbi.14290

[B5] Gentry AH (1993) A field guide to the families and genera of woody plants of northwest South America (Colombia, Ecuador, Perú), with supplementary notes on herbaceous taxa, Conservation International, Washington D.C., 1−895.

[B6] Hallé F Oldeman RAA Tomlinson PB (1978) Tropical trees and forests: an architectural analysis. Springer-Verlag, New York, 385 pp. 10.1007/978-3-642-81190-6

[B7] IUCN (2024) Guidelines for Using the IUCN Red List Categories and Criteria. Version 16. Prepared by the Standards and Petitions Committee. http://www.iucnredlist.org/documents/RedListGuidelines.pdf

[B8] Janovec PJ (2000) A systematic study of *Compsoneura* (Candolle) Warburg, a Neotropical member of the nutmeg family (Myristicaceae). PhD. thesis, Texas A&M University, College Station, Texas, 359 pp.

[B9] Janovec JP Harrison JS (2002) A morphological analysis of the *Compsoneurasprucei* complex (Myristicaceae), with a new combination for the Central American species *Compsoneuramexicana*. Systematic Botany 27(4): 662–673. http://www.jstor.org/stable/3093914

[B10] Janovec JP Neill AK (2003) Studies of the Myristicaceae: An overview of the *Compsoneuraatopa* complex, with descriptions of new species from Colombia. Brittonia 54(4): 251–261. 10.1663/0007-196X(2003)54[251:SOTMAO]2.0.CO;2

[B11] Jaramillo-Vivanco TS Balslev H (2001) Two new Myristicaceae from Ecuador. Nordic Journal of Botany 21(6): 561–566. 10.1111/j.1756-1051.2001.tb00809.x

[B12] Jaramillo-Vivanco TS Balslev H (2020) Revision of *Otoba* (Myristicaceae). Phytotaxa 441(2): 143–175. 10.11646/phytotaxa.441.2.3

[B13] Kühn U Kubitzki K (1993) Myristicaceae. In: Kubitzki K Rohwer J-G Bittrich V (Eds) The Families and Genera of Vascular Plants.Flowering Plants. Dicotyledons. Magnoliid, Hamamelid, and Caryophyllid families. Springer Verlag, Berlin, 457–467. https://doi. org/10.1007/978-3-662-02899-5_53

[B14] Paredes R Zárate-Gómez R Grandez-Rios JM (2023) *Compsoneuranallarettiana* (Myristicaceae), a new species from north-western Peru. Phytotaxa 592(2): 294–300. 10.11646/phytotaxa.592.3.7

[B15] Rodrigues WA Aymard CGA Berry PE (2001) Myristicaceae. In: Steyermark JA Berry PE Holst BK (Eds) Flora of the Venezuelan Guayana.Missouri Botanical Garden Press 6: 734–747.

[B16] Santamaría-Aguilar D Aguilar-Fernández R Lagomarsino LP (2019) A taxonomic synopsis of *Virola* (Myristicaceae) in Mesoamerica, including six new species. PhytoKeys 134: 1–82. 10.3897/phytokeys.134.37979 31686954 PMC6821834

[B17] Santamaría-Aguilar D Lagomarsino LP (2021) Two new species of *Otoba* (Myristicaceae) from Colombia. PhytoKeys 178: 147–170. 10.3897/phytokeys.178.64564 34140828 PMC8184734

[B18] Sasaki D (2009) [onwards] Neotropical Myristicaceae. In: Milliken W, Klitgaard B, Baracat A (Eds) Neotropikey - Interactive key and information resources for flowering plants of the Neotropics. http://www.kew.org/science/tropamerica/neotropikey/families/Myristicaceae.htm [accessed: 05 November 2023]

[B19] Sauquet H (2004) Systematic revision of Myristicaceae (Magnoliales) in Madagascar, with four new species of *Mauloutchia*. Botanical Journal of the Linnean Society 146(3): 146–351. 10.1111/j.1095-8339.2004.00343.x

[B20] Smith AC Woodhouse RP (1937) The American species of Myristicaceae. Brittonia 2: 393–510. 10.2307/2804799

[B21] Smith AC (1938) Compsoneura. Brittonia 2(5): 405–416. 10.2307/2804801

[B22] Smith AC (1950) Studies of South American plants. XII. Contributions from the United States National Herbarium 29(8): 318–320.

[B23] Smith AC (1956) Studies of South American plants. XV. American Journal of Botany 43(8): 573–574. 10.1002/j.1537-2197.1956.tb10536.x

[B24] Thiers BM (2024) [continuously updated] Index Herbariorum: A global directory of public herbaria and associated staff. New York Botanical Garden’s Virtual Herbarium. http://sweetgum.nybg.org/science/ih/ [Accessed 07.07.2024]

[B25] Vásquez-Martínez Soto-Shareva YC (2019) *Virolapseudosebifera* (Myristicaceae), una nueva especie de la selva alta del Perú. Rev. Q’euña 10(1): 07–12. 10.51343/rq.v10i1.314

[B26] Villanueva-Tamayo B Cogollo-Pacheco A (2024) A new species of *Compsoneura* (Myristicaceae) from interandean valleys of middle Magdalena and lower Cauca rivers, Colombia. Phytotaxa 637(3): 251–260. 10.11646/phytotaxa.637.3.3

[B27] Warburg O (1897) Monographie der Myristicaceen. Nova Acta Academiae Caesareae Leopoldino-Carolinae Germanicae Naturae Curiosorum 68: 1–680.

[B28] Warburg O (1905) Myristicaceae Costaricenses (Original diagnosen), Repertorium specierum novarum regni vegetabilis. Selbstverlag des Herausgebers, 71 pp. 10.1002/fedr.19050010503

[B29] Wilson TK (2004) Myristicaceae. In: Smith N Mori SA Henderson A Stevenson DW Heald SV (Eds) Flowering Plants of the Neotropics.New York Botanical Garden & Princeton University Press, Princeton, 261–262.

